# A Leucine Zipper Dimerization Strategy to Generate Soluble T Cell Receptors Using the *Escherichia coli* Expression System

**DOI:** 10.3390/cells11030312

**Published:** 2022-01-18

**Authors:** Angela Zhang, Alicja Piechocka-Trocha, Xiaolong Li, Bruce D. Walker

**Affiliations:** 1 Ragon Institute of MGH, MIT and Harvard, Cambridge, MA 02139, USA; azhang@mgh.harvard.edu (A.Z.); atrocha@mgh.harvard.edu (A.P.-T.); 2 Department of Medical Oncology, Dana-Farber Cancer Institute, Boston, MA 02115, USA; 3 Howard Hughes Medical Institute, Chevy Chase, MD 20815, USA; 4 Institute for Medical Engineering and Science (IMES) and Department of Biology, Massachusetts Institute of Technology, Cambridge, MA 02139, USA

**Keywords:** T cell receptor, protein engineering, leucine zipper, *E. coli* expression, refolding, purification

## Abstract

T cell-mediated adaptive immunity plays a key role in immunological surveillance and host control of infectious diseases. A better understanding of T cell receptor (TCR) recognition of pathogen-derived epitopes or cancer-associated neoantigens is the basis for developing T cell-based vaccines and immunotherapies. Studies on the interaction between soluble TCR α:β heterodimers and peptide-bound major histocompatibility complexes (pMHCs) inform underlying mechanisms driving TCR recognition, but not every isolated TCR can be prepared in soluble form for structural and functional studies using conventional methods. Here, taking a challenging HIV-specific TCR as a model, we designed a general leucine zipper (LZ) dimerization strategy for soluble TCR preparation using the *Escherichia coli* expression system. We report details of TCR construction, inclusion body expression and purification, and protein refolding and purification. Measurements of binding affinity between the TCR and its specific pMHC using surface plasmon resonance (SPR) verify its activity. We conclude that this is a feasible approach to produce challenging TCRs in soluble form, needed for studies related to T cell recognition.

## 1. Introduction

T lymphocytes are vital mediators of adaptive immunity. Each αβ T cell contains a unique αβ T cell receptor (TCR) that engages with antigens and initiates signaling transduction [1,2,3]. T cells develop in the thymus, where they undergo TCR β-selection, positive selection, and negative selection before migrating to peripheral lymphoid tissues [4,5,6,7]. T cells remain naïve until they have encountered their specific antigens in the form of a peptide-bound major histocompatibility complex (pMHC) on the surface of professional antigen-presenting cells such as dendritic cells [8] and macrophages [9]. TCR recognition of pMHC leads to proliferation and differentiation into either CD4^+^ or CD8^+^ effector T cells [8,10].

V(D)J recombination during T cell development generates a highly diverse repertoire of TCRα and TCRβ chains, needed for sampling polymorphic pMHCs [11,12,13,14]. TCR diversity is essential for providing the immune system with the ability to combat novel diseases [15]. For example, recent studies demonstrate the critical role of HIV-1-specific cellular immunity in maintaining spontaneous control of HIV without medications [16,17]. A deeper structural understanding of T cell recognition of pathogen-derived pMHC complexes is fundamental to both vaccine development and immunotherapeutic approaches for viruses and cancers, but this has been technically challenging [18,19].

Although some TCR and TCR-pMHC complex structures have been determined, many are difficult to obtain, and thus, answers to many biological questions remain elusive [1,20]. TCRα and TCRβ chains are both composed of two immunoglobulin (Ig) domains: a variable domain and a constant domain. Variable domains utilize six complementarity-determining region (CDR) loops to recognize antigen-bound MHC, in which germline-encoded CDR1 and CDR2 generally interact with MHCs, while non-germline encoded CDR3 is critical for discriminating antigens [21,22]. To understand and potentially augment these interactions, soluble TCR samples for 3-dimensional structures and binding affinity studies are needed.

Over the past three decades, several TCR sample preparation approaches have been developed, with two main conventional methods broadly used in the field. The most common method involves refolding recombinant TCRα and TCRβ chains as inclusion bodies in *Escherichia coli* (*E. coli*) by introducing a free cysteine residue in the constant domain of each TCR chain [23]. Briefly, the engineered TCRα and TCRβ inclusion bodies are denatured in denaturants such as urea or guanidine hydrochloride (guanidine-HCl) and then renatured in refolding buffer and dialyzed. Properly refolded dimers, comprised of α and β chains, can then be separated by multistep chromatography purifications [23]. A second conventional approach has been to express recombinant protein in mammalian cells for biophysical and functional studies by adding a pair of coiled-coil leucine zipper (LZ) sequences [24] to the C terminus of each TCRα and TCRβ chain [25]. However, not all TCRs can be refolded successfully in these manners (e.g., only one-third of TCRs in our lab).

The coiled-coil LZ has been shown to increase the success of TCR formation in mammalian cells [24,25]; yet these proteins are not well suited for most structural studies due to introduction of N- or O-glycans by mammalian expression systems [26,27]. However, glycosylation remains an important modification process as select proteins rely on glycans for protein stability, solubility, and activity. Notably, in the absence of glycans, glycosylation dependence can be rescued by a small number of local point mutations [28]. This is also observed when pair-wise cysteines are introduced for TCR engineering [23]. Here, we report an alternative method to produce soluble protein for challenging TCRs by applying a LZ dimerization strategy using the bacterial *E. coli* expression system. To demonstrate the utility of this approach, we use a TCR identified in our lab from a cytotoxic T lymphocyte (CTL) clone, designated C8, derived from an HIV elite controller expressing HLA-B*5701. Despite extensive efforts, this C8 TCR could not be successfully produced using conventional methods. We, therefore, proceeded by fusing a modified LZ sequence to the C terminus of the α and β chains of this HIV-1 specific TCR, designated C8-LZ. A Tobacco Etch Virus (TEV) protease cleavage site was inserted between each LZ and TCR α and β chain, allowing for a removable LZ for both biophysical and structural studies. We then performed protein-protein binding experiments to verify TCR activity.

## 2. Materials and Methods

### 2.1. Construct Design

The LZ sequence added to the TCRα C terminus is named LZ(+) (sequence: AQCKKKLQALKKKNAQLKWKLQALKKKLAQ). The LZ sequence added to the TCRβ C terminus is named LZ(−) (sequence: AQCEKELQALEKENAQLEWELQALEKELAQ).

### 2.2. DNA Synthesis and Plasmid Construction

TCRs were sequenced from T cell clones isolated from HIV-infected persons by pHLA tetramer staining. The tetramers were obtained from Dr. Søren Buus (University of Copenhagen, København, Denmark). DNA fragments encoding TCRα and TCRβ chains (codons optimized for *E. coli* expression) and DNA oligomers for polymerase chain reaction (PCR) were synthesized by GENEWIZ, Inc. (South Plainfield, NJ, USA). PCRs were performed using Q5 polymerase, restriction endonuclease, and T4 ligase (purchased from New England Biolabs, Inc., Ipswich, MA, USA). Gene fragments were inserted between *Nde I* and *Xho I* restriction sites of the pET22b(+) expression vector (purchased from Novagen, Inc., Beijing, China).

### 2.3. Inclusion Body Expression and Extraction

TCRα-pET22b(+) and TCRβ-pET22b(+) constructs were transformed into *E. coli* BL 21(DE3) competent cells (purchased from Invitrogen, Inc., Waltham, MA, USA) for protein expression. Transformed cells were cultured in Luria-Bertani (LB) medium (purchased from United States Biological, Inc., Salem, MA, USA). When cell density reached an OD_600_ value of ~0.8, the expression was induced with 1 mM IPTG at 37 °C for 4 h. Inclusion body pellets were then suspended and lysed via sonification in extraction buffer (50 mM Tris-HCl, 1 M NaCl, 2% Triton-X100, pH 9.0). Inclusion bodies were separated at 10,000 rpm for 10 min. To sufficiently lyse the cells and improve recombinant protein purity, inclusion bodies were suspended and lysed twice or more. Inclusion bodies were washed three times using wash buffer (50 mM Tris, 20 mM EDTA, pH 8.5). Purified inclusion bodies were solubilized in 6 M guanidine-HCl and stored at −20 or −80 °C.

### 2.4. Soluble TCR Refolding

20 mg TCRα and 30 mg TCRβ were mixed and rapidly diluted in 500 mL refolding buffer (100 mM Tris-HCl, 0.4 M arginine, 0.5 mM oxidized glutathione, 5 mM reduced glutathione, 2 mM EDTA, 4 M urea, 0.2 mM PMSF, pH 8.0). The resulting refolded protein solution was dialyzed twice against double distilled water (ddH_2_O) and twice against 10 mM Tris-HCl buffer (pH 8.0) using a 6–8 kD molecular weight cutoff dialysis membrane (purchased from Spectrum Labs, Inc., Rancho Dominguez, CA, USA).

### 2.5. Protein Purification and Examination

Dialyzed protein solution was filtered using a PES 0.22 μm membrane (purchased from Santa Cruz Biotechnology, Inc., Dallas, TX, USA), purified by nickel-NTA (Ni-NTA) (purchased from Qiagen, Inc., Hilden, Germany), and purified by gravity-flow chromatography, size exclusion chromatography (SEC) (products of GE Healthcare, Inc., Chicago, IL, USA). Refolded proteins at each step were evaluated by SDS-page gel. Protein concentration was measured by NanoDrop™ 2000 (product of ThermoFisher Scientific, Inc., Waltham, MA, USA).

### 2.6. Protein Digestion

To enzymatically cleave the LZ, the purified TCRαβ was mixed with homemade TEV protease [29] in 10 mL cleavage buffer (20 mM Tris-HCl, 200 mM NaCl, pH 8.0) in a TCR:TEV mass-ratio of 50:1 at 4 °C for over 24 h. The protein-enzyme mixture was gently resuspended every 8–10 h. Following TEV digestion of the LZ, 1 mL Ni-NTA resin (equilibrated with 20 mM Tris-HCl, 200 mM NaCl, pH 8.0) was added to the protein-enzyme mixture and resuspended at 4 °C to allow the Ni-NTA resin to bind His-tagged nontarget proteins. Reverse Ni-NTA gravity-flow chromatography was used to remove the 6xHis tagged LZ and TEV protease. Digested TCR, without a His tag, was collected in the flow-through and further purified by SEC.

### 2.7. Surface Plasmon Resonance

To examine soluble TCR activity, TCR and corresponding pHLA binding affinity was measured via Biacore S200 (product of GE Healthcare, Inc., Chicago, IL, USA). The biotinylated pHLA was coated on a Cytiva Series S sensor SA chip (purchased from Cytiva, Inc., Marlborough, MA, USA), and the refolded TCR served as the analyte flowed over the chip surface. Biotinylated pHLAs were obtained from Dr. Søren Buus (University of Copenhagen, København, Denmark).

### 2.8. Software for Data Analysis

Structural analysis was performed in Pymol (Version 2.4.1, Schrödinger, Inc., New York, NY, USA). Protein gel graphic figures were generated by ChemiDocTMTouch Imaging System (Bio-rad Laboratories, Inc., Hercules, CA, USA). Protein sequences were analyzed using Protein Identification and Analysis Tools on the ExPASy Server (https://web.expasy.org/protparam/). TCRs and pHLAs binding affinities were analyzed by Biacore Insight Evaluation Software(Biacore S200, Cytiva, Inc., Marlborough, MA, USA).

## 3. Results

### 3.1. TCRα and TCRβ Chain Construct Design and Protein Production Workflow

**Figure 1:**
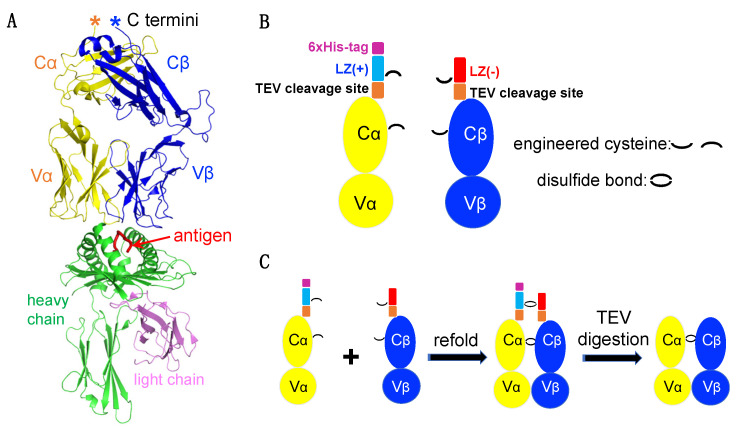
TCRαβ heterodimer construct design. (**A**) Overall structure of a representative TCRαβ-pMHC complex (PDB code: 1OGA). (**B**) Architectures of the engineered TCRα and TCRβ. (**C**) Schematic diagram depicting TCRαβ dimerization and TEV protease digestion.

Figure 1A is an example of a TCRαβ engaging with a pMHC in the highest resolution TCR-pMHC complex structure available (PDB, 1OGA), reported by the Yvonne Jones Lab at the University of Oxford (Oxford, United Kingdom) [27]. The yellow and blue ribbons represent the TCRα and TCRβ chains, respectively. The class I MHC heavy chain is shown in green and the beta-2 microglobulin (β2m) light chain in magenta. The antigenic peptide is represented by a red line lying in the antigen-binding domain of the heavy chain.

While soluble MHCs are relatively straightforward to prepare, producing soluble TCRs remains a major challenge. To overcome this bottleneck, a pair of charge complementary LZ sequences were added to the C-terminus of each TCRα and TCRβ chain (Figure 1A,B). Additionally, to facilitate protein purification, a 6xHis tag was added to the TCRα chain C terminal end, and a TEV protease cleavage sequence was inserted between the LZ and TCRα and β chains.

Engineered TCRα and TCRβ chains from the HIV-specific CTL clone C8 (a QW9-HLA-B57-tetramer sorted CTL clone) were expressed as inclusion bodies in *E. coli* and refolded into soluble protein. To increase TCRαβ stability, we introduced two free cysteine amino acids in each TCR chain, one within the LZ and the other within the constant domain of each TCRα and β chain (Figure 1B). Νotably, we also attempted to engineer an LZ construct without free cysteines but failed to produce our target protein. During proper refolding, four free cysteines form two inter-disulfide bonds to lock the dimer (Figure 1C). LZ charge complementarity on the TCRα and TCRβ facilitate TCRα-TCRβ heterodimer but prevent TCRα-TCRα and TCRβ-TCRβ dimerization. Homemade TEV protease was then used for digestion to remove the LZ (Figure 1C).

### 3.2. Inclusion Bodies Expression, Purification and Solubilization

**Figure 2:**
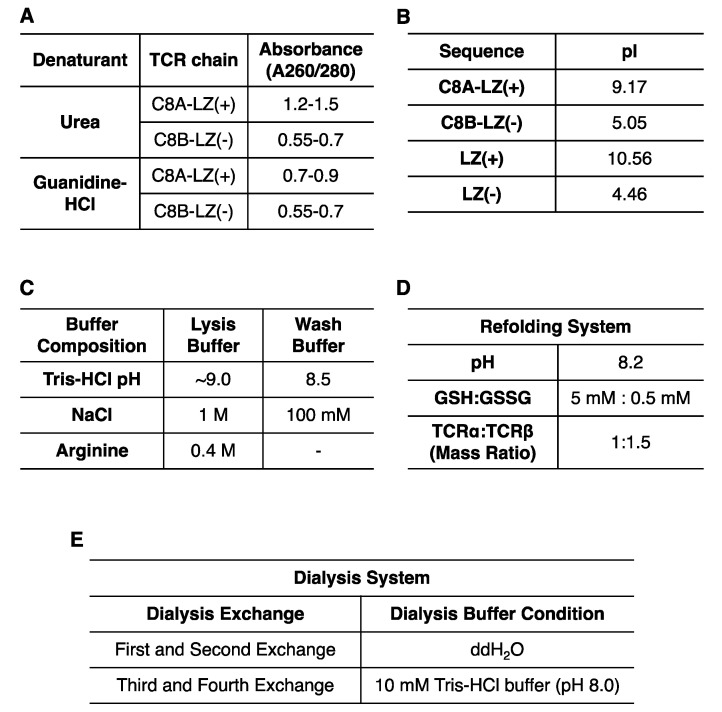
Optimization for TCR inclusion body expression, purification, and refolding. (**A**) Inclusion body purity varied with differing denaturants. (**B**) ProtParam measured pI values for each region of the refolded protein. (**C**) Composition of lysis buffer and wash buffer for TCR inclusion body purification. (**D**) Optimized TCRαβ refolding system. (**E**) Dialysis buffer conditions at each exchange.

A high yield of engineered C8 TCRα (termed C8A-LZ(+)) and TCRβ (termed C8B-LZ(−)) inclusion bodies were expressed in *E. coli*. The C8B-LZ(−) inclusion bodies fused with an acidic LZ sequence were purified and denatured easily by following general protocols. However, after applying the same extraction protocol to C8A-LZ(+) inclusion bodies, inclusion body pellets were difficult to solubilize using urea. Guanidine-HCl allowed for better solubility but consistently resulted in high A260/280 values, measured by NanoDrop^TM^ 2000 (Figure 2A). A pure protein sample A260/280 ratio is ~0.55, and a higher ratio may indicate contamination of isolated proteins with DNA [30]. This could result from *E. coli* extracted inclusion bodies or soluble proteins having a high isoelectric point (pI) due to numerous positively charged residues. Our alkaline LZ fused C8A-LZ(+) has a globally high pI of 9.17, and the LZ(+) itself possesses a pI of 10.56 (Figure 2B). These data suggested an optimized protocol was needed for C8A-LZ(+) inclusion body purification.

To address the concern of C8A-LZ(+) inclusion body DNA contamination, we combined multiple strategies (summarized in Figure 2C) to reduce DNA and inclusion body binding: (1) Lysis buffer and wash buffer pH was increased to lower protein surface charge; (2) Lysis buffer and wash buffer salt concentration was increased to improve ion strength and weaken DNA-protein interaction; (3) Arginine was added to lysis buffer as a DNA binding competitor; (4) Additional sonications were performed during lysis for increased separation of protein and DNA; (5) 6 M Guanidine hydrochloride was used to solubilize inclusion bodies. Utilizing these strategies, we observed much lower A260/280 for C8A-LZ(+) (Figure 2A).

Interestingly, we also found that a lower temperature can improve DNA contamination removal. Inclusion bodies solubilized in guanidine-HCl precipitate into white pellets after overnight storage at −20 °C. Proteins were then spun down at 12,000 rpm (4 °C) to remove pellets, and A260/280 of the supernatant reached ~0.7 (Figure 2A).

### 3.3. Refolding of Soluble C8 TCR

The refolding system for this challenging C8 TCR required optimization of several buffer components. We considered three important factors: pH, GSH:GSSG ratio, and C8A-LZ(+):C8B-LZ(−) ratio (Figure 2D). As reported by Coutard B. et al., acidic pI proteins prefer alkaline refolding buffers, while alkaline pI proteins prefer acidic refolding buffers [31]. The C8A-LZ(+) has a global pI of 9.17. On one hand, C8 TCRα and TCRβ without LZs have highly acidic pIs (Figure 2B) that optimally refold at pH 8.0; on the other hand, the two LZ sequences have complementary charges that facilitate C8 TCRαβ dimerization and require a pH closer to 7. Moreover, a relatively alkaline pH provides an oxidized environment for disulfide bond formation. Meanwhile, the ratio of GSH:GSSG was also optimized to promote proper disulfide bond formation. Lastly, based on the stability and solubility of each C8A-LZ(+) and C8B-LZ(−) chain, varying ratios of C8A-LZ(+):C8B-LZ(−) in the refolding system were tested as well.

Following experimentation, several conditions were optimized and summarized in Figure 2D,E. Refolded C8A-LZ(+):C8B-LZ(−) inclusion bodies were dialyzed in two manners: (i) A series of buffers with varying denaturant concentrations (ii) The same buffers without any denaturants. The yield of successfully refolded protein under each condition was identified by SDS-page gel. Our results indicated (1) a GSH:GSSG ratio of 5 mM:0.5 mM and a C8A-LZ(+):C8B-LZ(−) ratio of 1:1.5 were optimal (2) two dialysis exchanges with double distilled water (ddH_2_O) only followed by two exchanges with 10 mM Tris buffer (pH 8.0) were optimal for dialysis.

### 3.4. Soluble C8 TCR Purification

**Figure 3:**
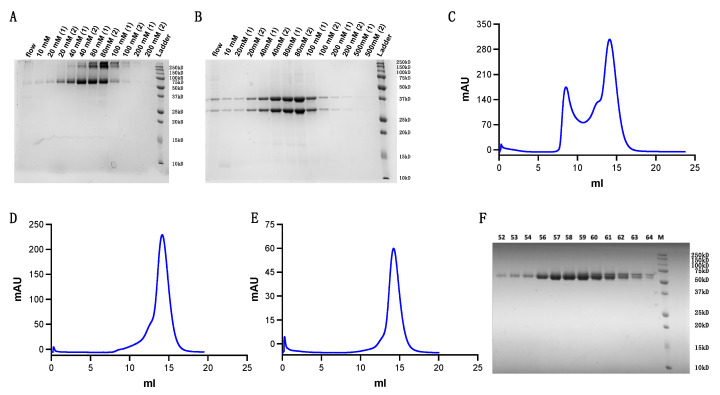
Refolded TCR purification. (**A**,**B**) Purity evaluation following Ni-NTA column purification at stepwise imidazole concentrations ranging from 10 mM to 200 mM. (**A**) Non-reducing SDS-page gel (**B**) Reducing SDS-page gel (**C**) First round of s200 Superdex SEC. (**D**) Second round of s200 Superdex SEC. (**E**) Third round of s200 Superdex SEC. (**F**) Purity examination of fractions eluted from the final round of SEC.

We scaled up our refolding volume to 1 L once our refolding system was optimized. To separate the soluble protein from the refolding system, we combined nickel affinity purification and SEC. We first concentrated our refolded protein from 1 L to 100 mL before incubating the protein for over 2 h with Ni-NTA Resin (purchased from Qiagen, Inc., Hilden, Germany). After washing and eluting the Ni-NTA resin with 10 mM to 200 mM imidazole at stepwise concentrations, we used SDS-page gels to identify the components of each sample fraction (Figure 3A,B).

Fractions containing target protein were merged and concentrated using 30 kD molecular weight cut-off centrifugal filters (purchased from MilliporeSigma, Inc., Burlington, MA, USA) to a small volume for s200 Superdex SEC (Figure 3C). Protein fractions were then concentrated and loaded for a second and third round of s200 Superdex SEC (Figure 3D,E). Results show the protein achieved high purity following three rounds of purification (Figure 3F).

### 3.5. C8-LZ TCR and QW9-B*57 Binding Affinity Measurements

**Figure 4:**
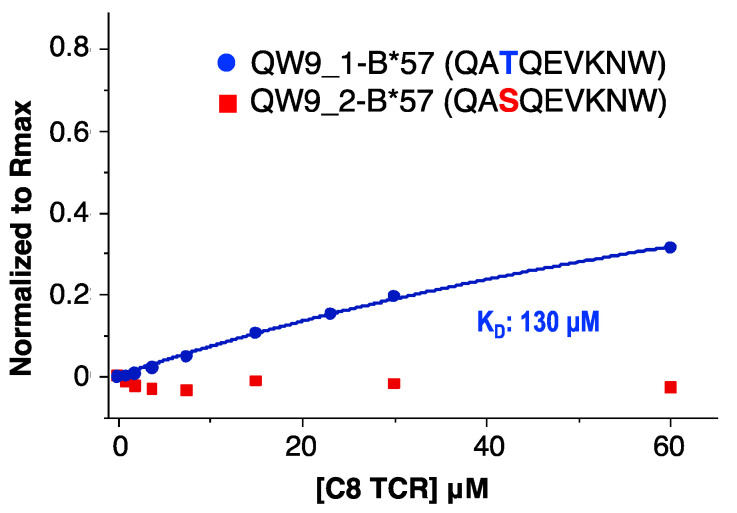
SPR binding affinity measurements for C8-LZ with QW9_1-B*57 and QW9_2-B*57. C8 TCR flowed through QW9_1-B*57 and QW9_2-B*57 coated chips (Series S sensor SA). (Blue curve represents QW9_1-B*57, KD: 130 μM; Red dots represent QW9_2-B*57, no detectable signaling).

To verify the functionality of our refolded C8-LZ, we tested its binding affinity with HLA-B*57, which presents the HIV Gag protein derived antigenic peptide QW9 (QW9_1). With a QW9 variant (QW9_2) bound to the same HLA-B*57 as a control, we performed surface plasmon resonance (SPR) to measure C8-LZ binding with QW9_1-B57 and QW9_2-B57. Using C8-LZ as the analyte, biotinylated QW9_1-HLA-B*57 and QW9_2- HLA-B*57 were coated on the same streptavidin sensor chip (Series S sensor SA). Refolded C8-LZ TCR had a measurable binding affinity with its specific peptide QW9_1 bound HLA-B*57 (Figure 4) with a dissociation constant of 130 μM, within the expected 1–200 μM range of ΤCR-pMHC binding [32]. However, no binding was detected for the QW9_2 variant bound HLA-B*57 (Figure 4). These results suggest that our refolding strategy can produce a competent TCR capable of specific binding to its cognate pMHC.

### 3.6. TEV Digestion for Leucine Zipper Removal

**Figure 5:**
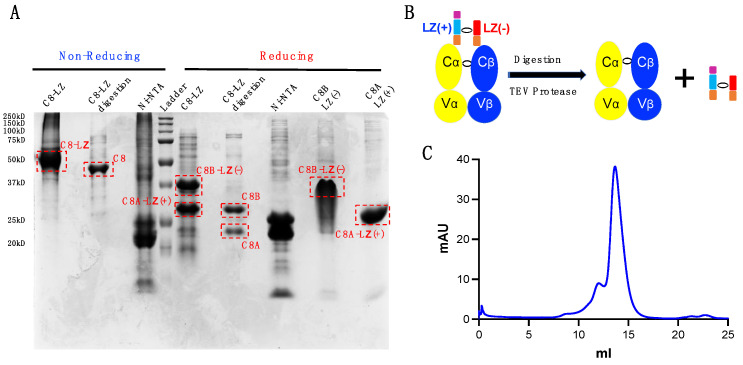
TEV digestion for LZ removal. (**A**) SDS-page gel examination for samples pre- and post-digestion. C8-LZ: pre-digested sample; C8-LZ digestion: digested C8 TCRαβ via reverse Ni-NTA column; Ni-NTA: post-digested proteins with a 6xHis tag bound to the reverse Ni-NTA column; C8A-LZ(+): solubilized C8A-LZ(+) inclusion body; C8B-LZ(−): solubilized C8B-LZ(−) inclusion body. (**B**) Schematic of TEV protease digestion. (**C**) s200 Superdex SEC separation of digested C8 protein.

Samples for crystallization require high purity and homogeneity. However, an LZ at the TCRαβ C-terminus could affect molecule packing for growing TCR or TCR-pMHC complex crystals [33]. To address this issue, we inserted a TEV protease cleavage site between the LZ and TCRαβ. We proceeded by using homemade 6xHis tagged TEV protease. Digested TCRαβ was then separated using a reverse Ni-NTA column. The LZ-6xHis, undigested TCR, and TEV protease could then bind the Ni-NTA column, while digested TCRαβ flowed through the column at a low imidazole concentration (Figure 5A). Figure 5B depicts our digestion workflow. Furthermore, additional purification can be done via SEC. Figure 5C shows our digested C8 TCRαβ purified via s200 Superdex SEC.

### 3.7. Application to TCRs beyond C8 TCR

**Figure 6:**
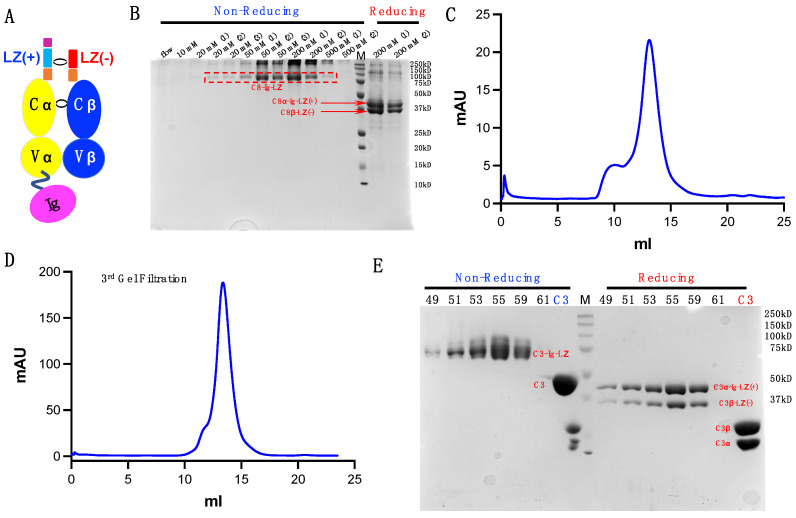
Additional TCRs produced using our dimerization strategy. (**A**) Modified TCR and TCR-Ig constructs. (**B**) C8-Ig from Ni-NTA column elution at stepwise imidazole concentrations. (**C**) C8-Ig elution from s200 Superdex. (**D**) Final round of s200 SEC for C3-Ig. (**E**) SDS-gel examining C3-Ig purity following the final round of s200 Superdex SEC.

To determine if our proposed strategy can be applied to TCRs beyond C8 TCR, we examined two additional TCR constructs, C8-Ig and C3-Ig, in which the TCRα chain was modified with an extra Ig fold domain (Figure 6A). C8 is the same previously described TCR, and C3 is another HIV-specific TCR. This TCR-Ig construct design comes from one of our ongoing cross-link study projects, in which the Ig-fold domain is a target of another protein derived domain. Despite unsuccessful refolding of C8-Ig and C3-Ig TCRs using conventional approaches, the LZ strategy efficiently facilitated the refolding of these two TCRs. Following our optimized refolding and purification protocol described previously, we achieved high yields of these TCRs. Taking C8-Ig-LZ as an example, Ni-NTA column purification is similar to purification for C8-LZ (Figure 6B). Figure 6C displays s200 Superdex SEC for C8-Ig. Similarly, for C3-Ig-LZ, Figure 6D shows the result following the third-round of s200 Superdex SEC. C3-Ig-LZ protein fractions, alongside a conventionally refolded and purified C3 TCR for comparison, were examined by SDS-page gel (Figure 6E). These results suggest that our engineered LZ for dimerization can serve as a general strategy for preparing soluble TCRs.

## 4. Discussion

To obtain soluble TCRs for structural, biophysical, and functional studies, we developed a general LZ dimerization strategy for challenging TCR production. Our biochemical data show that this strategy can generate soluble TCR α:β heterodimers using the *E. coli* expression system. Furthermore, our SPR data using a soluble C8 TCR show measurable binding with its specific pMHC while binding was abrogated by the same MHC presenting an epitope variant. This is consistent with sophisticated TCR-pMHC specificity interactions [34]. Moreover, the LZ can be removed using TEV protease, making the digested TCR more suitable for structural studies, particularly for crystallography by reducing potential artifacts [33]. Of note, strategies for extracting TRA-LZ(+) inclusion bodies may be significant for the preparation of inclusion bodies or soluble forms of other alkaline proteins.

Given TCR recognition of an antigen presented by an MHC molecule is the critical first step for T cell function activation [8,10], detailed information regarding TCR-pMHC interactions is key to addressing basic immunological questions such as T cell cross-reactivity and viral immune escape [35] and can direct the development of immunotherapies such as TCR-like CARs targeting neoepitopes [36] and bispecific antibodies targeting both TCR and pMHC [37,38]. CAR-T cells are often designed by fusing T cell signaling transduction elements with an antibody’s single-chain variable fragment (scFv), targeting a cancer biomarker [39]. However, neoantigens and viral epitopes presented by MHC molecules on cancer cells and infected cells pose as alternative targets. Therefore, a potent pMHC-specific TCR poses as a promising strategy for CAR-T or TCR-T cell therapy [40,41].

**Figure 7:**
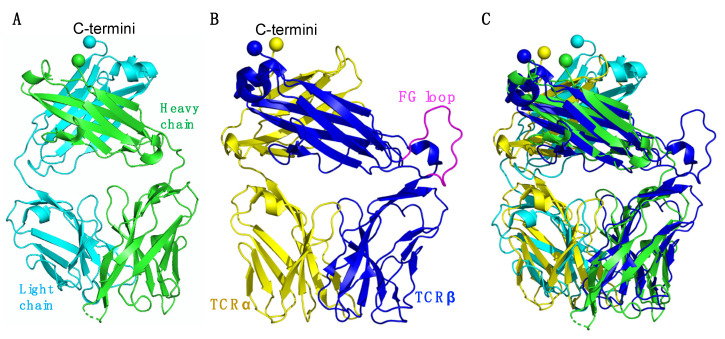
Structural comparison of the Fab and TCR. (**A**) Overall view of a Fab (PDB code: 4XAW). The Fab heavy chain is shown in green and the light chain in cyan. (**B**) Overall view of a TCR (PDB code: 1OGA). The TCRα chain is shown in yellow, the heavy chain in blue, and the TCRβ chain FG loop is shown in magenta. (**C**) Structural alignment of Fab and a TCR. (**A**–**C**) C-terminal residues of each chain are displayed as spheres.

The antigen-binding fragment (Fab) region of an antibody has a highly similar 3-dimensional architecture to TCRs, composed of Ig domains [42], as shown using representative examples in Figure 7A,B. However, unlike a TCR with an FG loop in the F and G β strands, Fab does not contain such a loop region (Figure 7B). Interestingly, when superimposed, the Fab and TCR C termini share highly similar geometry (Figure 7C). In order to produce soluble Fabs, gene fragments are typically integrated into an IgG framework and expressed in mammalian cells [43]. Fabs can then be digested from purified IgG; however, this process is costly. Based on the structural similarity of Fab and TCR, our LZ dimerization strategy for refolding may be explored as an approachable and economical way to produce Fabs for structural and therapeutic studies.

Future directions of our ongoing work focus on structural and functional related experiments for C8 while also testing more TCRs to further assess the success rate of our approach. Our strategy provides an option for producing challenging soluble TCRs, such as our reported C8, C8-Ig and C3-Ig, that cannot be produced using conventional methods. Based on our long-term work on various TCRs, we propose that for a newly isolated TCR, the conventional method using cysteine engineered constant domains of TCRα and TCRβ for *E. coli* expression should be attempted first [23]. If unsuccessful, our present method should be attempted next, before the mammalian expression system as this final method introduces the potential of post-translational modifications and high-cost related concerns. The advantage to our LZ dimerization strategy using the *E. Coli* system is that it is an all-around feasible method that is low-cost, time-efficient, and suitable for structural studies. Our approach builds upon the conventional *E. coli* expression system strategy to allow for the soluble production of challenging TCRs, needed for studies related to T cell recognition.

## Data Availability

The data presented in this study are available on request from the corresponding author.
